# A Stepwise Diagnostic Approach to Transmural Myocardial Infarction With Non-obstructive Coronary Arteries With Microvascular Obstruction Highlighting the Role of Cardiac MRI in Suspected Thrombolysis

**DOI:** 10.7759/cureus.85489

**Published:** 2025-06-06

**Authors:** Kyle E Thurmann, Pallavi Bellamkonda, Olga M Kalinkin

**Affiliations:** 1 Medicine, Creighton University School of Medicine, Phoenix, USA; 2 Cardiology, St. Joseph's Hospital and Medical Center, Phoenix, USA; 3 Radiology, St. Joseph's Hospital and Medical Center, Phoenix, USA

**Keywords:** cardiac mri, coronary angiography, diagnostic imaging, microvascular obstruction, minoca, myocardial infarction, spontaneous thrombolysis, thrombophilia

## Abstract

Myocardial infarction with non-obstructive coronary arteries (MINOCA) presents a diagnostic challenge due to its heterogeneous etiologies and the absence of obstructive coronary lesions. We report the case of a 74-year-old male with hypertension, prior deep vein thrombosis, and a history suggestive of thrombophilia under evaluation, on chronic warfarin therapy, who presented with chest pain, ST elevation, and elevated troponins. Coronary angiography revealed no obstructive coronary artery disease. Cardiac magnetic resonance imaging (MRI) demonstrated an acute transmural myocardial infarction with microvascular obstruction in the left anterior descending artery region. Alternative cardiac and extracardiac causes were excluded, and the diagnosis was revised to MINOCA. This case underscores the diagnostic power of cardiac MRI and suggests spontaneous thrombolysis as a plausible mechanism, based on the transmural infarction pattern, presence of microvascular obstruction, and absence of angiographic obstruction.

## Introduction

Myocardial infarction (MI) with non-obstructive coronary arteries (MINOCA) accounts for 5%-7% of all acute MI cases, with a median age of 55 years and a disproportionate prevalence in women. Approximately 40% of MINOCA patients are female, which is a notably higher proportion than observed in obstructive MI [1]. Despite the absence of obstructive lesions, these patients remain at significant risk for adverse cardiovascular outcomes. MINOCA represents a heterogeneous group of conditions defined by the universal criteria for acute MI, including a ≥20% change in cardiac troponin with at least one value above the 99th percentile and clinical evidence of ischemia through symptoms, electrocardiogram (ECG) changes, or imaging. Additionally, MINOCA requires the absence of obstructive coronary artery disease (major epicardial vessel stenosis ≤50%) on angiography and no overt non-cardiac cause for the presentation, such as sepsis, pulmonary embolism, or aortic dissection [2,3].

The diagnostic uncertainty surrounding MINOCA poses challenges for timely management and prognostication. While conventional coronary angiography remains essential in the initial workup of acute MI, its limitations in detecting dynamic coronary events, microvascular dysfunction, or subtle plaque disruption highlight the need for advanced imaging [4]. Cardiac MRI, in particular, has emerged as a key modality for distinguishing ischemic from non-ischemic myocardial injury. In 2019, the American Heart Association emphasized the importance of comprehensive diagnostic evaluation in MINOCA and recommended cardiac MRI as a central tool in etiologic assessment [4].

We present a rare case of MINOCA in which conventional coronary angiography revealed no obstructive atherosclerosis, while cardiac MRI demonstrated an acute transmural MI with microvascular obstruction (MVO) in the left anterior descending (LAD) artery region. MVO, also known as the “no-reflow phenomenon,” is an area of the coronary microvasculature that does not allow reperfusion, reflecting irreversible microvascular damage. It occurs despite successful recanalization of the epicardial artery and is associated with larger infarct size, adverse ventricular remodeling, and worse functional recovery [5]. A 2024 prospective cohort study of 198 patients with ischemic MINOCA found that MVO was detected by cardiac MRI in 1.5% of cases and was associated with an elevated risk of adverse outcomes [6]. Notably, the patient was receiving chronic warfarin therapy for a suspected but unconfirmed thrombophilic disorder. These findings are most suggestive of spontaneous thrombolysis, a phenomenon in which an occlusive thrombus resolves before angiographic evaluation, observed in an estimated 0.1% of angiography-proven thrombotic occlusions based on published case report data [7].

## Case presentation

A 74-year-old male former smoker with a history of hypertension and left lower extremity deep venous thrombosis presented to an outside hospital with acute, non-radiating midsternal chest pain, rated 2/10 in severity. Although mild in intensity, the pain was persistent and appropriately prompted an ECG, which revealed ST-elevation in the lateral leads with reciprocal ST-depression in leads II, III, and aVF. Troponin I was elevated at 11.5 ng/mL (normal <0.04 ng/mL). These findings led to a timely acute coronary syndrome (ACS) evaluation. He had no prior history of coronary artery disease or heart failure and was on chronic warfarin therapy for suspected hereditary thrombophilia. The patient was transferred to our facility for coronary angiography and reperfusion evaluation. Upon transfer, he remained hemodynamically stable with improved chest pain while on nitroglycerin and heparin drips, and coronary angiography was scheduled for the following morning.

Twelve hours after admission, his condition worsened, with elevated temperature, heart rate, respiratory rate, and blood pressure, while oxygen saturation remained normal (Table 1). Although no neurological or renal involvement was documented, the ongoing myocardial injury, evidenced by progressive elevation in troponin I, supported the diagnosis of hypertensive emergency based on cardiac end-organ damage. Troponin I levels peaked at 38.478 ng/mL. He was treated with anticoagulation, vasodilators, analgesia, and antihypertensives to manage both the suspected ST-elevation MI (STEMI) and hypertensive emergency. A summary of administered medications, including dosages, routes, and indications, is provided in Table 2. Following treatment, vital signs improved, with normalization trends in blood pressure, heart rate, and respiratory rate, while oxygen saturation remained stable and temperature unchanged (Table 3). Although the patient’s clinical deterioration occurred 12 hours after admission, angiography had already been scheduled for the following morning due to initial hemodynamic stability and timely cardiology evaluation upon transfer. Thus, the interval before catheterization was not due to a delay, but rather a clinically appropriate management decision. The working diagnosis at that time was STEMI, suspected to be secondary to coronary artery disease and complicated by hypertensive emergency.

**Table 1:** Selected vital signs recorded 12 hours after hospital admission

Vital Sign	Observed Value	Reference Range	Interpretation
Temperature	38.1°C	36.1-37.2°C	Increased
Heart rate	114 bpm	60-100 bpm	Increased
Respiratory rate	37 breaths/min	12-20 breaths/min	Increased
Blood pressure	190/101 mmHg	<120/80 mmHg	Increased
Oxygen saturation (SpO₂)	95%	95-100%	Normal

**Table 2:** Medications administered in the emergency setting for acute chest pain and severe hypertension

Medication	Dose	Route	Frequency	Indication
Heparin	25,000 units	Intravenous	Continuous infusion	Prevent clot formation
Nitroglycerin	20 mcg/min	Intravenous	Continuous infusion	Lower blood pressure and chest pain
Nitroglycerin	0.4 mg	Sublingual	Single dose	Relieve chest pain
Aspirin	81 mg	Oral	Daily	Prevent blood clots
Morphine	2 mg	Intravenous	Every two hours as needed	Relieve chest pain
Hydralazine	10 mg	Intravenous	Every four hours	Lower blood pressure

**Table 3:** Selected vital signs following initial medical treatment for acute chest pain and severe hypertension

Vital Sign	Observed Value	Reference Range	Interpretation
Blood pressure	140/80 mmHg	<120/80 mmHg	Improved but elevated
Heart rate	101 bpm	60-100 bpm	Slightly elevated
Respiratory rate	26 breaths/min	12-20 breaths/min	Elevated
Oxygen saturation (SpO₂)	95%	95-100%	Normal
Temperature	38.1°C	36.1-37.2°C	Increased

**Figure 1:**
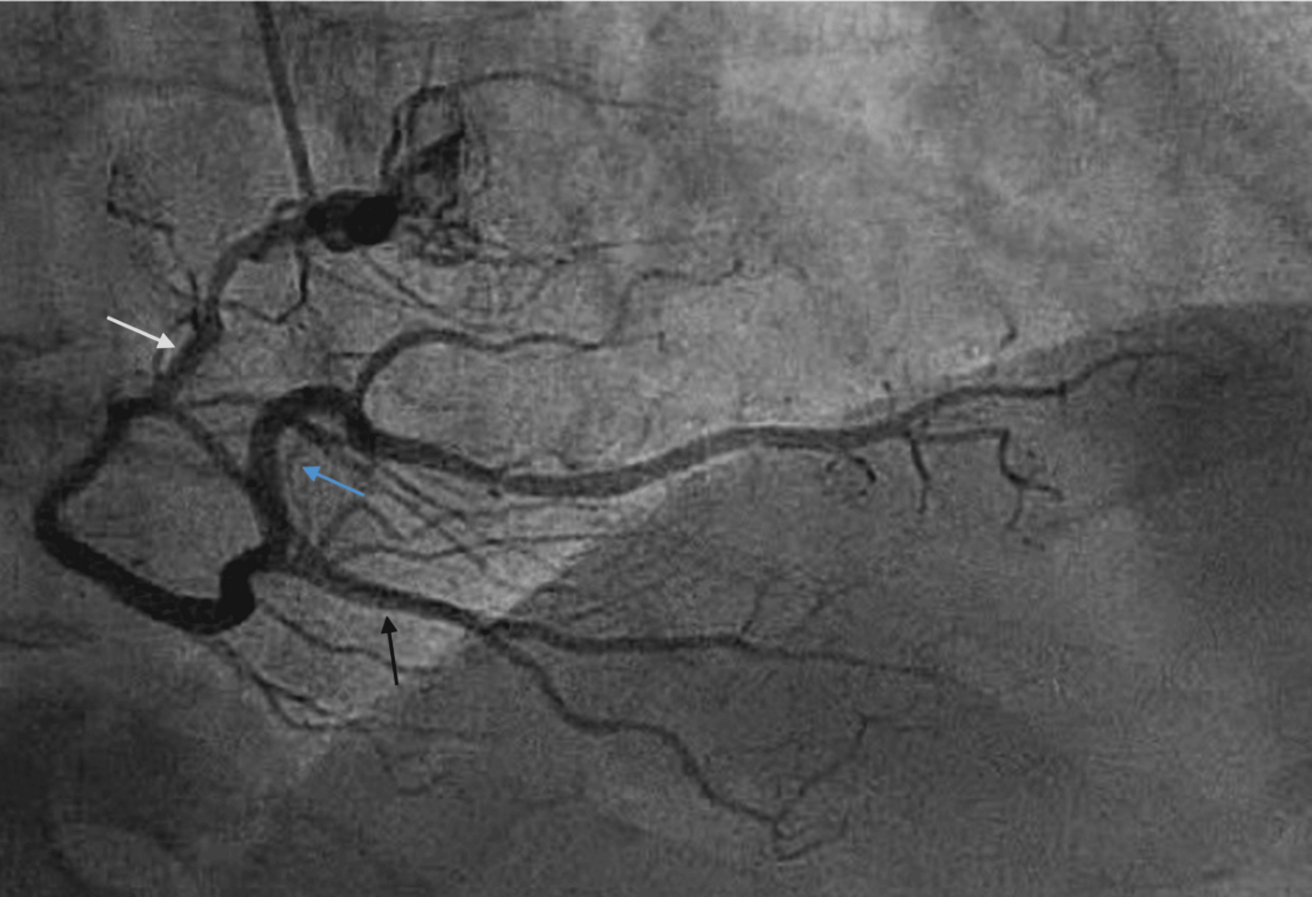
Right coronary artery angiography (right anterior oblique view) Right anterior oblique projection of the right coronary system demonstrating the right coronary artery (white arrow) giving rise to the posterior descending artery (black arrow) and the posterior left ventricular branch (blue arrow). No hemodynamically significant lesions are observed in the right coronary system.

**Figure 2:**
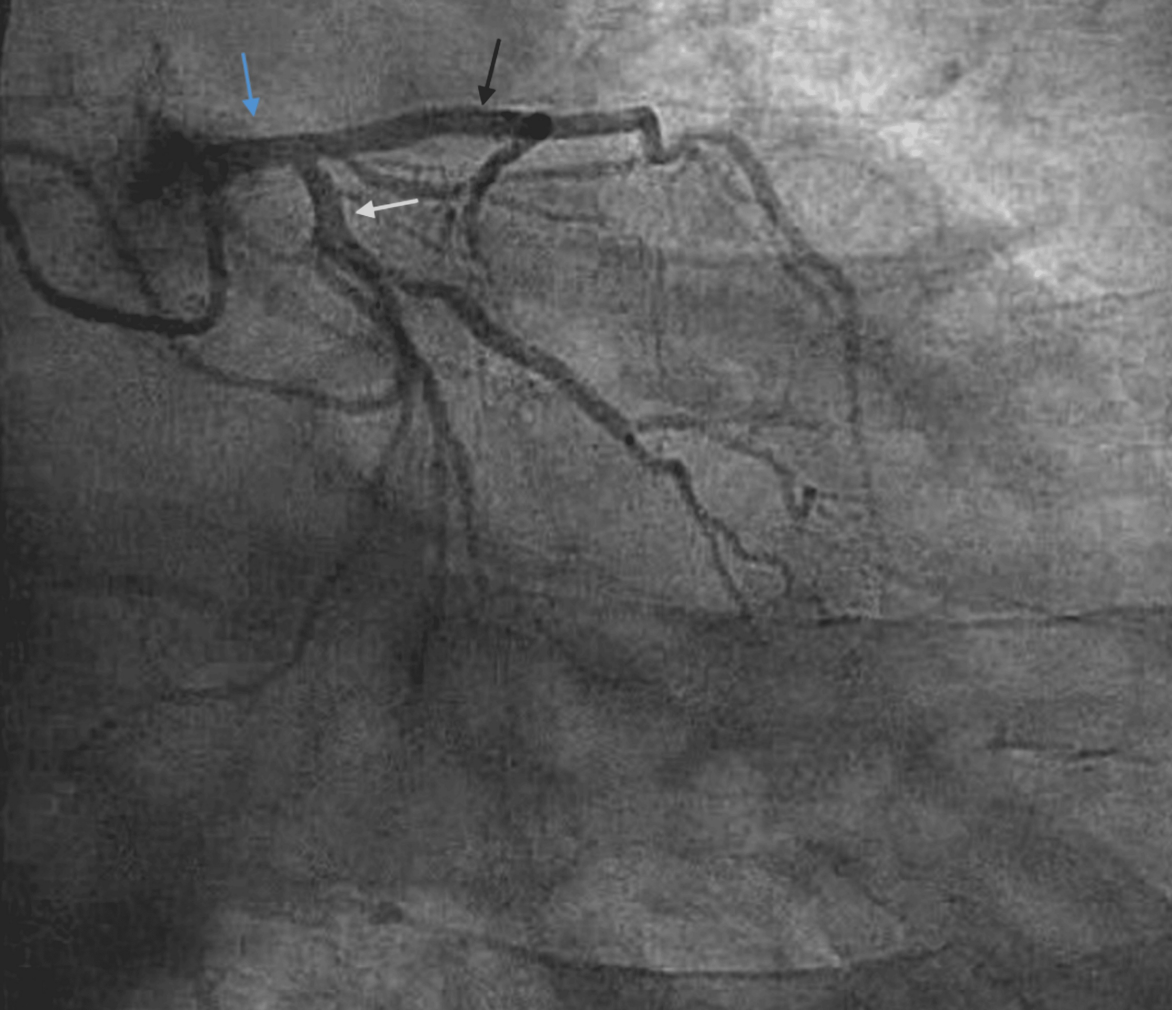
Left coronary artery angiography (left anterior oblique view) Left anterior oblique projection of the left coronary system demonstrating the bifurcation of the left main coronary artery (blue arrow) into the left anterior descending (black arrow) and left circumflex (white arrow) arteries. No hemodynamically significant lesions are observed in the left coronary system.

Coronary angiography revealed a right-dominant coronary system with moderate (50-60%) stenosis in the mid-right coronary artery. However, the fractional flow reserve (FFR) was 0.95, indicating that the lesion was not causing ischemia (FFR ≥ 0.80 is considered hemodynamically insignificant). The LAD and left circumflex arteries showed only minimal luminal irregularities, and no percutaneous coronary intervention was performed. Angiographic images of the coronary system are presented in Figures 1, 2.

Given the absence of obstructive coronary artery disease despite clinical and biochemical evidence of MI, the diagnosis was revised to troponin-positive non-obstructive coronary arteries (TpNOCA), a broader working term that describes patients with elevated cardiac biomarkers and non-obstructive coronary arteries prior to establishing an ischemic versus non-ischemic etiology. TpNOCA includes both ischemic (e.g., MINOCA) and non-ischemic causes (e.g., myocarditis).

**Figure 3:**
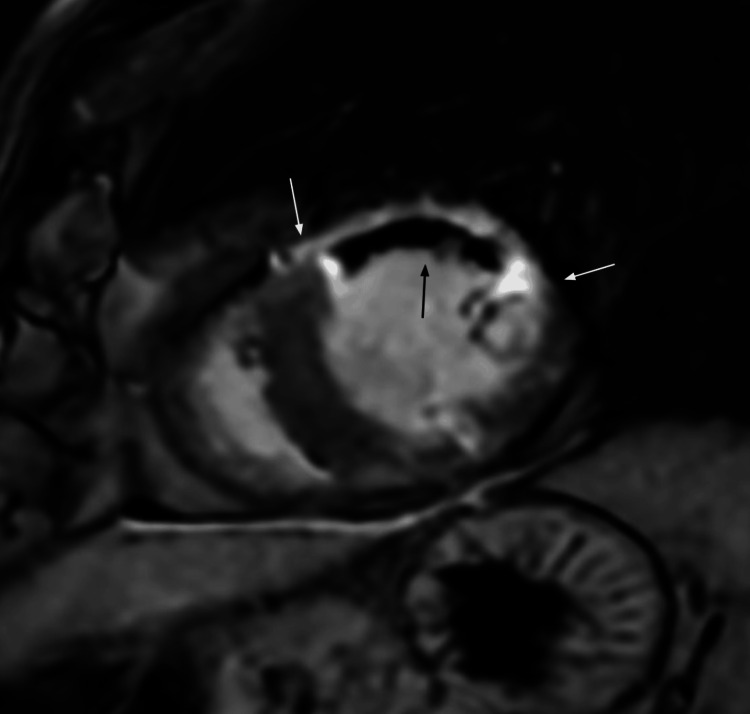
Short-axis view of the heart with late gadolinium enhancement There is high signal intensity of late gadolinium enhancement involving the entire anterior wall of the mid left ventricle, consistent with the transmural myocardial infarction (white arrows). The large region of low signal intensity in the subendocardial and mid-myocardial zone in the transmural infarct core zone is a no-reflow region (microvascular obstruction) sign of the acute myocardial infarction (black arrow).

**Figure 4:**
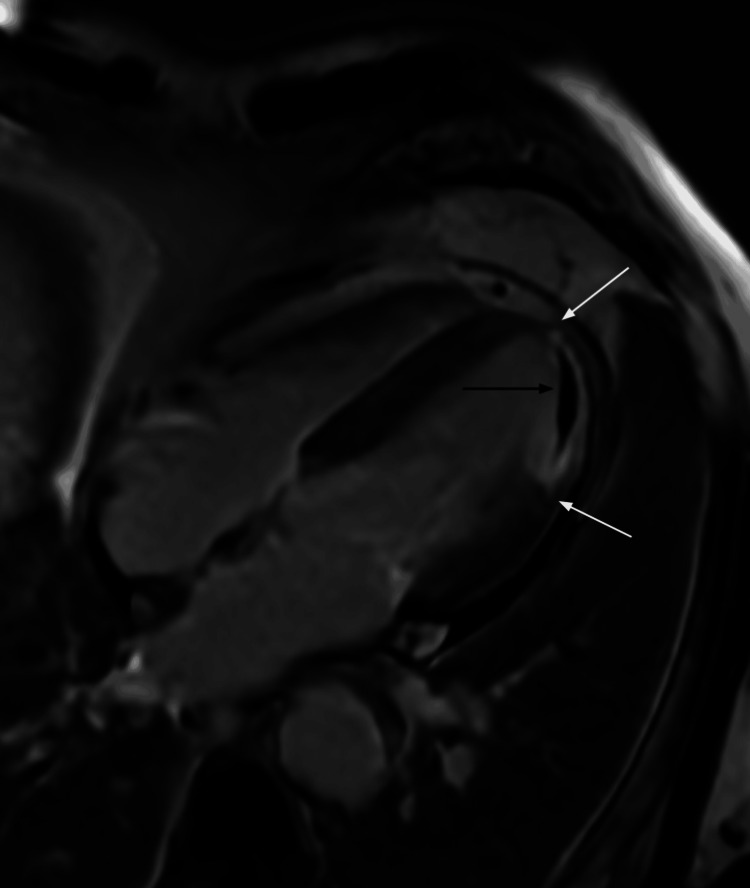
Four-chamber view of the heart with late gadolinium enhancement There is high signal intensity of late gadolinium enhancement involving the entire lateral wall of the left ventricular apex, consistent with the transmural myocardial infarction (white arrows). The large region of low signal intensity in the subendocardial and mid-myocardial zone of the transmural infarct is a no-reflow region (microvascular obstruction) sign of the acute myocardial infarction (black arrow).

Cardiac MRI was performed and revealed transmural late gadolinium enhancement (LGE), indicating infarcted myocardium, involving the mid and apical anterior-lateral segments, along with a central region of low signal intensity (hypoenhancement) in the subendocardial and mid-myocardial zones, consistent with MVO (Figures 3, 4). These findings corresponded to regional wall motion abnormalities ranging from hypokinesis to akinesis of the mid anterolateral left ventricular wall. Left ventricular ejection fraction was reduced to 38%. No myocardial edema or other imaging features suggestive of myopericarditis were observed. These findings supported a final diagnosis of MINOCA with MVO in the LAD region. MINOCA specifically refers to an ischemic mechanism within the broader category of TpNOCA that fulfills the universal definition of MI.

The patient was discharged in stable condition with a new medication regimen focused on cardiovascular risk reduction, including atorvastatin, lisinopril, metoprolol succinate, and spironolactone. Warfarin was continued due to his suspected but unconfirmed hereditary thrombophilia, and clopidogrel was added for antiplatelet therapy. Dual antiplatelet therapy with aspirin was not pursued given the patient’s anticoagulated state and concern for bleeding risk. He was advised to follow up with his primary care provider within one to two weeks and to return to the emergency department if symptoms recurred.

## Discussion

The diagnosis of MINOCA remains a descriptive, exclusion-based process that requires a stepwise evaluation [3]. In this case, the patient’s initial presentation was suggestive of ACS. In accordance with the 2021 American Heart Association/American College of Cardiology guidelines for ACS evaluation, prompt ECG and troponin testing were performed, confirming STEMI with significantly elevated troponin levels [8]. These findings warranted invasive coronary angiography, which demonstrated non-obstructed coronary arteries. Given the clinical and biochemical evidence of MI in the absence of obstructive coronary artery disease, the diagnosis was revised to TpNOCA, a working diagnosis encompassing both ischemic and non-ischemic causes of myocardial injury [9,10].

Cardiac MRI subsequently revealed imaging features consistent with an acute transmural MI and MVO, including transmural LGE, subendocardial no-reflow, regional wall motion abnormalities, and reduced ejection fraction. These findings pointed toward an ischemic mechanism and thus supported reclassification to MINOCA, following the exclusion of non-ischemic cardiac conditions (e.g., myocarditis, heart failure) and extracardiac causes (e.g., pulmonary embolism, aortic dissection). Although vasospasm and spontaneous coronary artery dissection cannot be definitively excluded in the absence of provocative testing or intracoronary imaging, the infarct pattern on MRI was more consistent with a thrombotic event than with these alternative mechanisms.

According to the contemporary diagnostic framework, MINOCA is defined by the universal criteria for MI, absence of obstructive epicardial coronary artery stenosis (>50%), and no clear alternative non-ischemic explanation at initial evaluation [3]. Among MINOCA cases, the presence of transmural LGE in the LAD region and MVO is associated with worse outcomes, despite angiographically normal epicardial vessels [2,11].

This case raises an important clinical question: how can a transmural infarction with MVO develop in the absence of coronary obstruction? The cardiac MRI findings, including regional wall motion abnormalities, reduced ejection fraction, transmural LGE, and subendocardial no-reflow, suggest an ischemic mechanism, most commonly associated with plaque rupture, coronary embolism, or spontaneous coronary artery dissection [9].

In this case, spontaneous thrombolysis of a ruptured coronary plaque is suspected. However, the precise mechanism remains unclear, and this hypothesis remains speculative in the absence of confirmatory intracoronary imaging or histopathologic evidence. One possibility involves the patient’s chronic warfarin use for suspected but unconfirmed thrombophilia, which may have contributed to endogenous thrombolysis, although the role of warfarin in promoting arterial thrombus resolution remains uncertain and less well established compared to its effects on venous thromboembolism [12]. Another theory relates to plaque thrombus phenotype. White thrombi, which are platelet-rich with dense fibrin networks, are more amenable to spontaneous lysis due to smaller size and shorter ischemic times. In contrast, red thrombi, composed primarily of fibrin and red blood cells, are larger and more resistant to lysis [13-15]. However, this distinction remains hypothetical in our case, as no intracoronary imaging or histopathologic confirmation of thrombus composition was obtained.

Alternatively, coronary embolism remains a plausible mechanism. Inherited thrombophilias increase the risk of both venous and arterial thrombotic events, including coronary embolism. A paradoxical embolism via a patent foramen ovale (PFO) or a left atrial thrombus could temporarily occlude a coronary artery before spontaneously resolving through endogenous fibrinolysis, particularly in an anticoagulated patient [16]. However, in this case, no echocardiographic evaluation (e.g., transthoracic or transesophageal echocardiogram with bubble study) was performed to assess for a PFO or intracardiac thrombus, limiting our ability to definitively rule out an embolic source.

MINOCA carries a substantial long-term mortality risk, with estimates ranging from 5-10% at one year and up to 16% at five years, depending on the underlying etiology [17,18]. Emerging evidence suggests that prognosis differs based on the mechanism, with ischemic MINOCA, particularly cases with cardiac MRI evidence of infarction, associated with worse outcomes than non-ischemic causes such as myocarditis or takotsubo cardiomyopathy [18]. In this case, cardiac MRI was essential in identifying a transmural infarct pattern with MVO, confirming an ischemic etiology. These findings not only excluded alternative diagnoses but also guided post-discharge management. Specifically, the confirmation of MI supported initiation of statin therapy, ACE inhibition, beta-blockade, and single antiplatelet therapy with clopidogrel. Dual antiplatelet therapy with aspirin was not pursued due to the patient’s anticoagulated state and elevated bleeding risk.

## Conclusions

This case illustrates a rare presentation of MINOCA characterized by transmural STEMI and MVO in the absence of obstructive coronary artery disease. Spontaneous thrombolysis of a coronary thrombus is proposed as a plausible mechanism, potentially influenced by the patient’s long-term warfarin therapy for suspected but unconfirmed hereditary thrombophilia. However, this remains speculative, particularly in the absence of intravascular imaging or confirmatory workup for embolic sources. This case further underscores the importance of a systematic, stepwise diagnostic approach, beginning with clinical assessment and exclusion of non-ischemic causes, followed by coronary angiography and advanced imaging such as cardiac MRI, with recognition that modalities like intracoronary imaging or provocative testing may also help identify culprit lesions when available. Cardiac MRI played a pivotal role in confirming the ischemic etiology and identifying MVO, reinforcing its diagnostic utility in MINOCA. The MRI-confirmed infarction with MVO guided the initiation of secondary prevention therapy with statins, ACE inhibitors, beta-blockers, and single antiplatelet therapy, while avoiding dual antiplatelet therapy due to bleeding risk. Recognizing such presentations is essential for accurate diagnosis, risk stratification, and personalized management. Broader study of similar MINOCA cases is warranted to further refine diagnostic pathways and therapeutic strategies.
